# Patent relatedness and velocity in the Chinese pharmaceutical industry: A dataset of Jaccard similarity indices

**DOI:** 10.1016/j.dib.2021.106814

**Published:** 2021-02-04

**Authors:** Charlotte Marie Vorreuther, Thierry Warin

**Affiliations:** a CIRANO (Inter-University Research Center on the Analysis of Organizations), 1130, Sherbrooke West, Suite 1400, Montreal H3A 2 M8, QC, Canada; b Department of International Business, HEC Montréal, 3000, chemin de la Côte-Sainte-Catherine, Montreal H3T 2A7, QC, Canada

**Keywords:** Pharmaceutical industry, Patents, Derwent world patents index, Jaccard similarity indice, Natural language processing, Innovation, China

## Abstract

The dataset is about innovation dynamics in the pharmaceutical industry in China. Innovation dynamics is interpreted as knowledge transfer across technologies and through time (velocity). The dataset provides access to 143,916 Jaccard similarity indices. A Jaccard similarity indice is a distance measure between two units. Here, they proxy relatedness across technologies (classes) and through time (velocity). The Jaccard similarity indices are computed based on a Natural Language Processing treatment of 69,923 patents in the pharmaceutical industry in China from 1990 to 2017.

## Specifications Table

**Table:** 

Subject	Social sciences
Specific subject area	Innovation
Type of data	Table, Figure, CSV file
How data were acquired	Source data extracted from Derwent World Patent Index (DWPI)
Data format	Raw, Analyzed
Parameters for data collection	Patents from the top 20 classes (4-digit level) about the pharmaceutical innovation in China
Description of data collection	Using “pharmaceutical” as a keyword, algorithmic collection of patents corresponding to the top 20 classes from DWPI
Data source location	Primary data source: DWPI (https://clarivate.com/derwent/dwpi-reference-center/dwpi-manual-code/)
Data accessibility	The Data are made available in the supplementary material coming with this article or through an R package on Github (see below). Repository name: Github Direct URL to data: https://github.com/warint/innovation_pharma_china Instructions for accessing these data: In R, type the function: library(innovation_pharma_china)

## Value of the Data

• The Jaccard similarity indices computed in this dataset allow us to map patents' relatedness in the Chinese pharmaceutical industry. The Jaccard similarity provides a cross-section relatedness and longitudinal cartography.
• Researchers can benefit from these data to capture the actual dynamics of innovation, with a particular interest in knowledge creation and relatedness in China's pharmaceutical industry. The data can also benefit policymakers and firms for the same reason.
• Researchers in innovation can use the similarity indices as either a feature or a target variable in their models. As a feature variable, the similarity indices can help describe the success of some public policies, firms, or innovation ecosystems. As a target variable, they may help find the source of innovation dynamics in the Chinese pharmaceutical industry.

## 1 Data Description

The dataset is about innovation dynamics in the pharmaceutical industry in China. Innovation dynamics is interpreted as knowledge transfer across technologies and through time (velocity). Knowledge transfer across technologies is here proxied by similarity across patent classes, and knowledge transfer velocity is proxied by similarity from one class during one year to the same class or another class in a different year. The dataset presented in this article consists of Jaccard similarity coefficients. They result from a Natural Language Processing treatment of 69,923 patents in the pharmaceutical industry in China from 1990 to 2017. The dataset comprises 143,916 Jaccard similarity coefficients. These coefficients are computed between 4-digit classes of patents in the Chinese pharmaceutical sector. We consider the top 20 (4-digit) classes for the overall period of interest from 1990 to 2017. The top-20 filter is computed based on the number of patent descriptions. Overall, for the 20 classes from 1990 to 2017, we have considered 69,923 patent descriptions.

These patent descriptions have been grouped by 4-digit classes and tokenized. All the features in this dataset result from a series of transformations of the original data from the Derwent World Patents Index (DWPI). The various features in the dataset are computed indicators based on the data and the source patents' metadata. One of these treatments was a Natural Language Processing-based treatment, which consisted of creating a standardized framework for the compilation of the Jaccard similarity indices. The process, the code, and some examples of descriptive statistics are accessible on the GitHub repository https://github.com/warint/innovation_pharma_china.

More precisely, we provide:

1. an overview of the methodology and some functionalities on the following Github repository address: https://warint.github.io/innovation_pharma_china/,
2. access to some of the code to retrieve and visualize the raw data at the following address: https://github.com/warint/innovation_pharma_china/blob/main/R/ipcR.R,
3. access to the raw data here: https://github.com/warint/innovation_pharma_china/tree/main/data,
4. access to an R package archived on Github through the following command in R: “devtools::install_github(“warint/innovation_pharma_china”).”

Having access to the data through an R package allows greater integration with a researcher's workflow. Indeed, the R package loads directly in the researcher's statistical environment. The researcher can then directly perform the required analysis.

The next table describes the different variables of the data file shared with this article.

Table 2 describes the top 20 classes at the 4-digit level.

**Table 1:** Description of the dataset variables.

Variable name	Description
name_i	corresponds to class_i + year_i containing all patents of this group and year in the dataset
id_i	identification for patent group i
class_i	manual code section for patent group i
year_i	year of patent filing
vol_i	number of patents in the group (name_i)
name_j	corresponds to class_j + year_j containing all patents of this group and year in the dataset
id_j	identification for patent group j
class_j	manual code section for patent group j
year_j	year of patent filing for group j
vol_j	number of patents in the group (name_j)
id_p	combination of id_i and id_j
class	coded in 1 for same class and in 0 when different class
year_diff	the difference in years between year_i and year_j of patent filing
vol_diff	difference in the amount of patents filed in each class for a respective year (difference vol_i and vol_j)
Jaccard_Similarity	similarity between patent class i and patent class j and their respective years

**Table 2:** Description of the top 20 manual code sections (1990–2017).

Manual code sections	Category	Sub-category
B14-N	Pharmaceutical activities	Organs
D05-H	Fermentation industry	Microbiology, laboratory procedures
B04-A	Natural products (or genetically engineered), polymers	Alkaloids, plant extracts
B14-S	Pharmaceutical activities	Miscellaneous activity terms
B14-F	Pharmaceutical activities	Drugs acting on the blood and cardiovascular system
B04-C	Natural products (or genetically engineered), polymers	Polymers
B14-C	Pharmaceutical activities	Anaesthetics and drugs relieving fever, inflammation and pain
B04-E	Natural products (or genetically engineered), polymers	Nucleic acids
B12-M	Diagnostics and formulation types	Formulations type
B14-J	Pharmaceutical activities	Drugs acting on the muscular and nervous systems
B14-H	Pharmaceutical activities	Cancer related drugs
B11-C	Process, apparatus	General process, apparatus
B14-A	Pharmaceutical activities	Antimicobials
B14-E	Pharmaceutical activities	Drugs acting on the gastrointestinal system
B10-A	Aromatics and cycloaliphatics (mono and bicyclic only), aliphatics	Rarer chemical groups general
B14-G	Pharmaceutical activities	Drugs acting on the immune system
B14-D	Pharmaceutical activities	Hormonal, antihormonal, enzyme inhibitors
A12-V	Polymer applications	Medical, dental, cosmetics and veterinary
B07-D	Heterocyclics, mononuclear	Sole hetero(s) nitrogen
B06-D	Heterocyclic fused ring	Sole hetero(s) nitrogen

## 2 Experimental Design, Materials and Methods

Patents have been collected through the Derwent World Patents Index (DWPI) database. It covers 14.3 million inventions from 40 worldwide patent-issuing authorities.

Using the “pharmaceut*” keyword, we initially collected 238,870 patents from 1990 to 2017 (retrieved October 18th, 2017), of which 69,923 were filed at the SIPO in China. The Chinese patents were downloaded in .html and .txt formats, and they encompassed all the industry, product and process innovations.

The patents were merged into a first dataset, which contained the full patent description with title, abstract, publication date, assignees, inventors, etc.

**Fig. 1:**
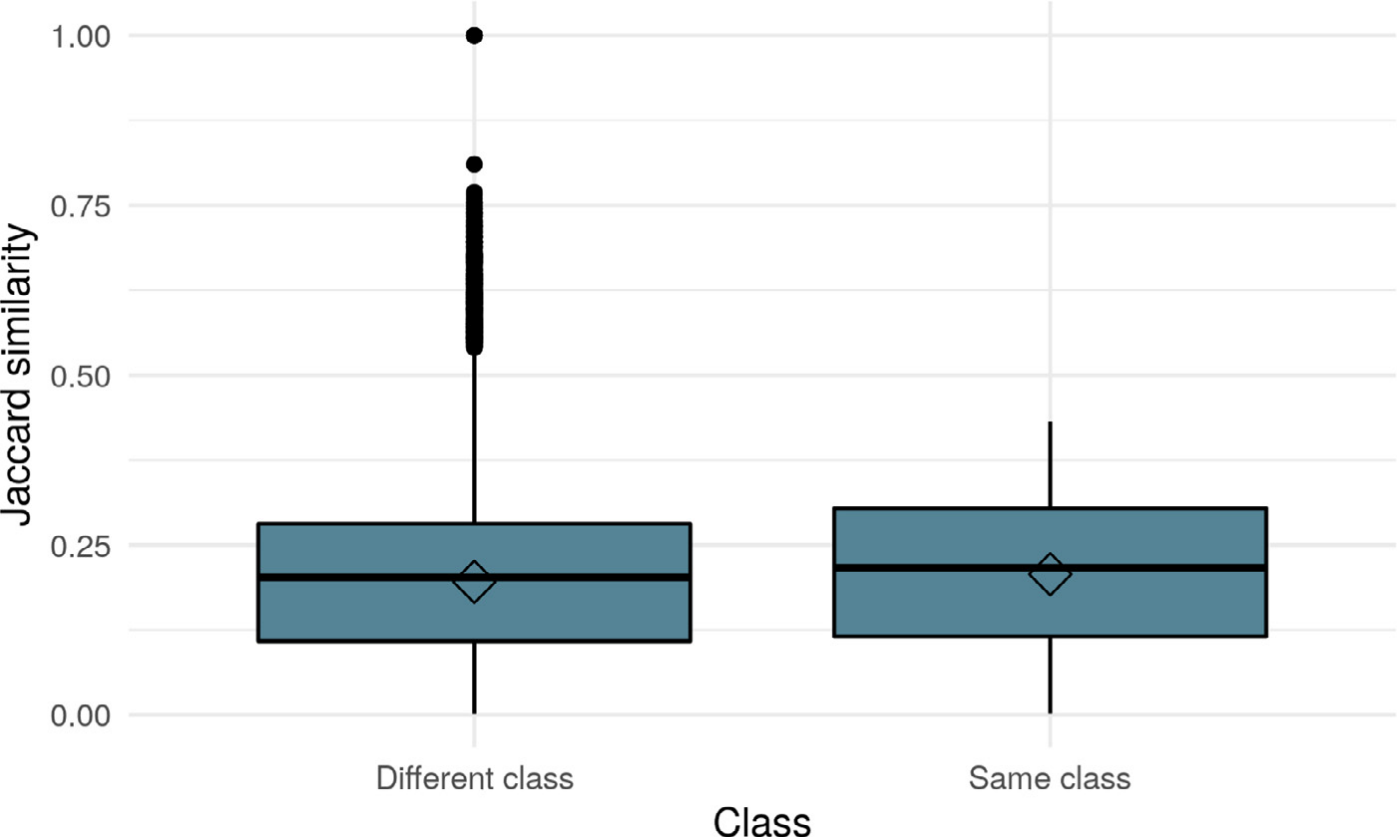
Distribution of Jaccard similarity for different/same classes.

The dataset was further cleaned in a subsequent step to consider only patent applications and not granted patents. Patent applications include changes to the patent content as well as petty patents. Then, willing to look at the technology's innovation dynamics, we grouped the patents by narrow patent classes at the 4-digit level. We considered the top 20 classes (see Fig. 1). We provide a boxplot perspective in order to illustrate the interquartile range (IQR), the median and the outliers. The latter are potentially interesting observations for some research questions interested in their high level of similarity.

As patents can belong to multiple classes, and as there may not be a patent in a specific narrow class during a year, we ended up with 537 classes__year combinations, leading to the computation of 143,916 (537 × 536/2) Jaccard similarity indices.

To compute the Jaccard similarity indices, we proceeded to a Natural Language Processing-based analysis of the patents, which requires access to some high-power-computing servers. We thus combine structured with unstructured data, extracting knowledge and information [1]. Unstructured data of a patent are texts, such as claims, abstracts or descriptions of the invention whose information is lost when analyzing solely structured data such as patent number, filing data and assignees [2].

For each year, we pasted each patent's descriptions in a class, creating one single text. After removing stop words (such as section titles), we proceeded to the tokenization for each class and each year. All computation involving the tokenization of the descriptions is based on Silge and Robinson [3], using the tidytext package [4] and the tidyverse package [5] in R. Text mining used in the patent analysis is “largely based on NLP, property-function based approaches, neural network-based approaches, and semantic-based approaches” [6]. We decided to use the n-gram technique. The patents' texts have been split into 5-character gram components, following Alschner [7]. This approach's advantage is that it retains the word order in contrast to bag-of-words approaches that measure the word occurrence. When analyzing patents, it is beneficial to take the word order into account due to the descriptions' scientific nature. Pharmaceutical components might occur in different contexts. Thus, a bag-of-words approach would skew the results. Also, in this NLP stage, DWPI is an interesting database for it has the advantage of providing global patent data in English. The translation has been undertaken by industry experts, thereby ensuring the accurate translation of the content, which is crucial for the tokenization.

After splitting the text into 5-character gram components, text similarity was calculated using Jaccard's formula (Jaccard, 1908).

$$S_{ij}=\frac{\left|A_{i}\cap A_{j}\right|}{\left|A_{i}\cup A_{j}\right|}$$

After the NLP stage, we created a dictionary of these tokens. We then proceeded to measuring the similarity across all the different classes and through time, based on this dictionary, as documented in the cluster analysis literature.

**Fig. 2:**
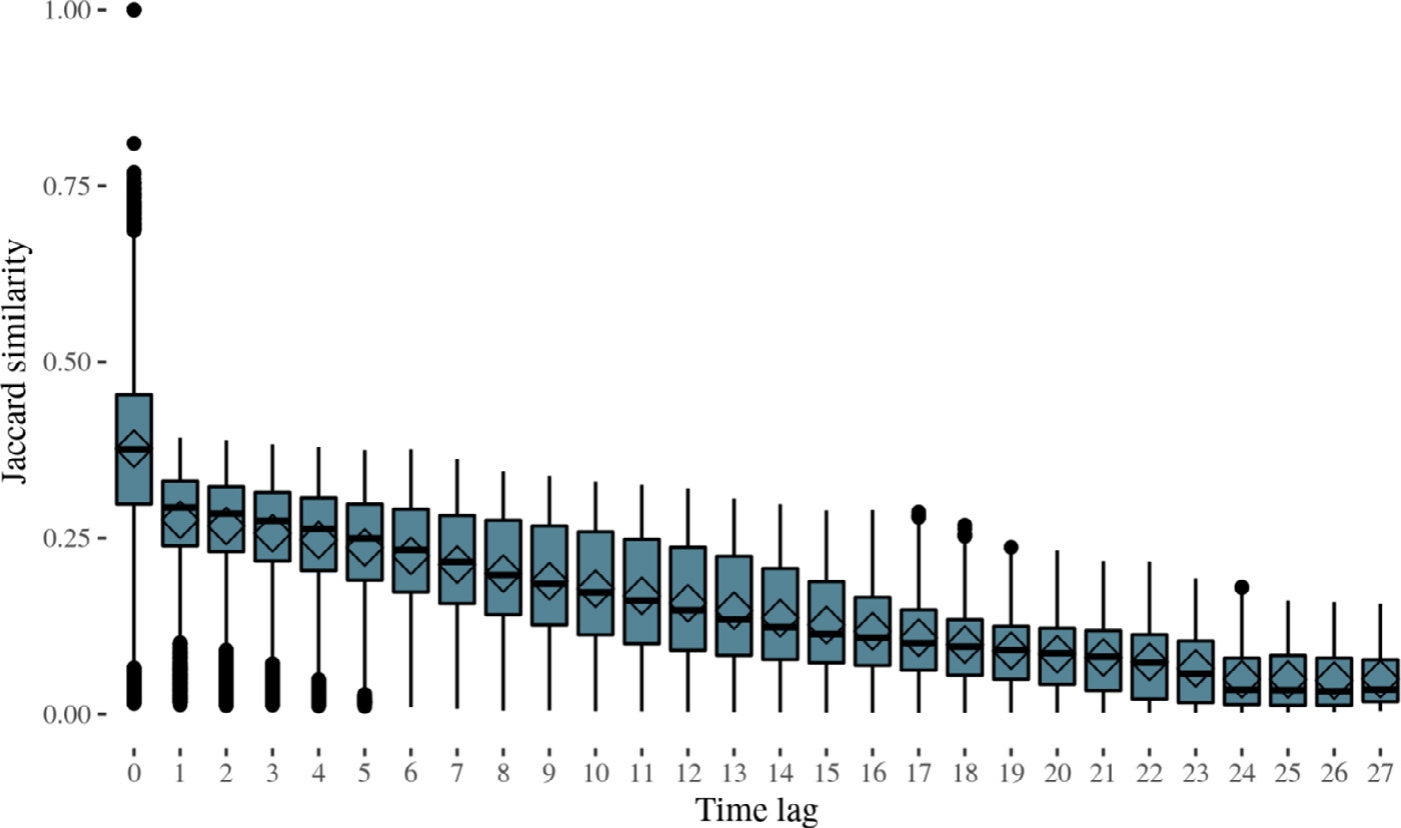
Distribution of Jaccard similarity of all classes through time.

To conclude, the text-as-data approach - and more specifically, the Jaccard similarity coefficient used in this article - allows measuring the text-similarity across classes and through time (see Figs. 1 and 2). Fig. 2 illustrates the cross-similarity of a class with all the patent classes in previous years. Thus, it shows the yearly stock of patents that are used in the current classes.

## Ethics Statement

No human or animal subjects were involved. The authors have the right to distribute the new data generated by their algorithms based on the data obtained from DWPI.

## CRediT Author Statement

**Vorreuther:** Data curation, Methodology, Investigation; **Warin:** Conceptualization, Visualization, Writing- Reviewing and Editing.

## Declaration of Competing Interest

The authors declare that they have no known competing financial interests or personal relationships which have or could be perceived to have influenced the work reported in this article.
